# Primary hydatid of psoas muscle

**DOI:** 10.4103/0974-7796.65112

**Published:** 2010

**Authors:** Imtiaz Wani, Rauf A. Wani, Khusrheed Alam Wani

**Affiliations:** Department of General Surgery, SKIMS, Srinagar, Kashmir, India

Sir,

A 23-year-old female presented with painless right lower abdominal swelling of two-month duration. There was no history of any trauma, fever or any cattle contacts. General physical examination was unremarkable. Abdominal examination on deep palpation revealed a swelling which was firm, nontender, globular, noncompressible with negative cough impulse in right lower abdomen.

**Figure 1:**
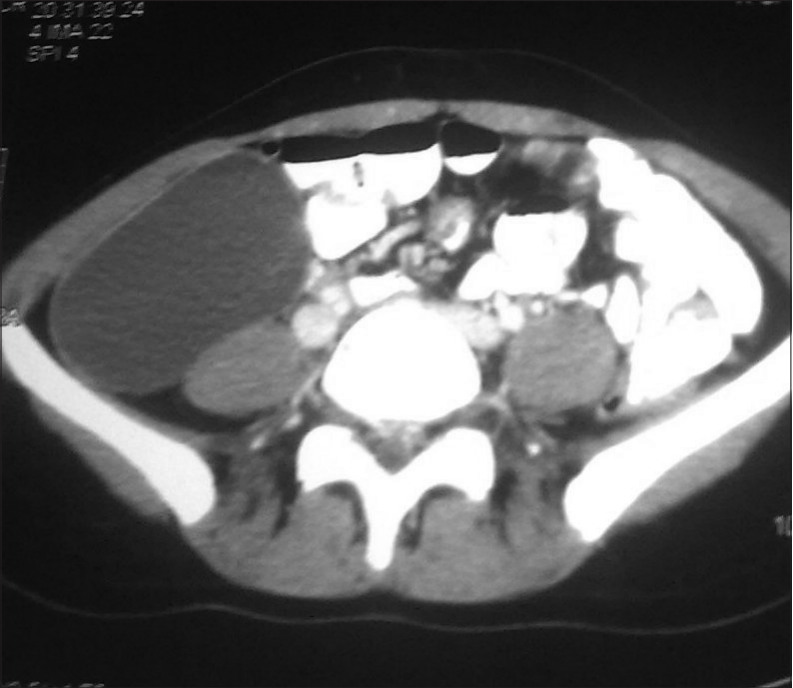
Contrast enhanced CT scan showing unilocular swelling arising from psoas muscle cystic mass in dimensions measuring 19.4×11×11.6 cm^3^ suggestive of hydatid cyst

Ultrasonography abdomen revealed a cystic unilocular swelling arising from right psoas muscle. Abdomino-pelvic computed tomography scan showed a cystic swelling measuring 19.4 × 11 ×11.6 cm^3^ arising from right psoas muscle and was suggestive of hydatid cyst [Figure 1]. There was no other evidence of hydatid disease on computed tomography of abdomen and chest. Hydatid serology was negative. By extraperitoneal approach via right iliac fossa region, total cystectomy was done. Post operative period was uneventful. Patient was discharged with four weeks on albendazole therapy with no recurrence seen in follow-up.

Isolated retroperitoneal location of hydatid cyst has been reported to be very exceptional, even in endemic areas.[1] Location in the muscular tissue accounts for 2–3% of all cases in body.[2] Psoas muscle is an unusual location for hydatid cyst accounting for only 1 to 3% of cases and can be unilateral or bilateral. Hydatid of psoas muscle can be isolated or associated with hydatid disease elsewhere in body. Hydatid disease in the muscles progresses slowly and is rarely life-threatening. Because of location, hydatid of psoas muscle usually remains asymptomatic and is found incidentally or an enlarging cyst can compress adjacent structures; kidney leading to its nonfunctioning, ureter, or the vertebrae. Hydatid serology in confirming diagnosis is often seen negative.

A high suspicion of clinical index is prime in suspecting hydatid psoas in endemic areas. Ultrasonography, computed tomography and the magnetic resonance imaging delineating soft tissue extent are sufficient for diagnosis. Psoas abscess, retroperitoneal cystic tumours and tuberculosis are common differential diagnosis. Intramuscular infestation may mimic a soft tissue tumor leading to inappropriate cyst rupture with the attendant risks of anaphylaxis and dissemination to other organs, so a preoperative correct diagnosis is important.

Preoperatively percutaneous aspiration drainage and the medical therapy are done to reduce size of the cyst. In order to prevent intraperitoneal dissemination of cyst extraperitoneal approach is preferred. Treatment involves partial cystectomy or the total cystectomy depending on the muscular extent of disease.

Hydatid of psoas muscle is uncommon to see. It can present as abdominal swelling. Surgery is preferred modality of treatment.
